# AbSet: A Standardized Data Set of Antibody Structures for Machine Learning Applications

**DOI:** 10.1021/acs.jcim.5c00410

**Published:** 2025-05-11

**Authors:** Diego S. Almeida, Matheus V. Almeida, Jean V. Sampaio, Eduardo M. Gaieta, Andrielly H. S. Costa, Francisco F. A. Rabelo, César L. Cavalcante, Geraldo R. Sartori, João H. M. Silva

**Affiliations:** † Laboratory of Structural and Functional Biology Applied to Biopharmaceuticals, 611310 Fundação Oswaldo Cruz, Fiocruz Ceará, Eusébio 61773-270, Brazil; ‡ Instituto Oswaldo Cruz, Fiocruz, Rio de Janeiro, Rio de Janeiro 21040-900, Brazil; § 28121 Universidade Federal do Ceará, Fortaleza 60020-181, Brazil; ∥ Pasteur-Fiocruz Center on Immunology and Immunotherapy, 37903 Fundação Oswaldo Cruz, Fiocruz Ceará, Eusébio 61760-000, Brazil

## Abstract

Machine learning algorithms have played a fundamental role in the development of therapeutic antibodies by being trained on data sets of sequences and/or structures. However, structural data sets remain limited, especially those that include antibody–antigen complexes. Additionally, many of the available structures are not standardized, and antibody-specific databases often do not provide molecular descriptors that could enhance ML models. To address this gap, we introduce AbSet, a curated dataset comprising over 800,000 antibody structures and corresponding molecular descriptors, including both experimentally determined and in silico-generated antibody–antigen complexes. We systematically retrieved antibody structures from the Protein Data Bank (PDB), applied rigorous standardization protocols, and expanded the dataset through large-scale protein–protein docking to generate structural variants of antibody–antigen interactions. Each model was classified as high, medium, acceptable, or incorrect quality based on structural similarity to reference experimental complexes. This classification enables both the construction of a decoy set of confirmed non-binders and the generation of high-confidence augmented structural data for machine learning applications. AbSet is publicly available via the Zenodo repository, with accompanying scripts hosted on GitHub (https://github.com/SFBBGroup/AbSet.git).

## Graphical abstract

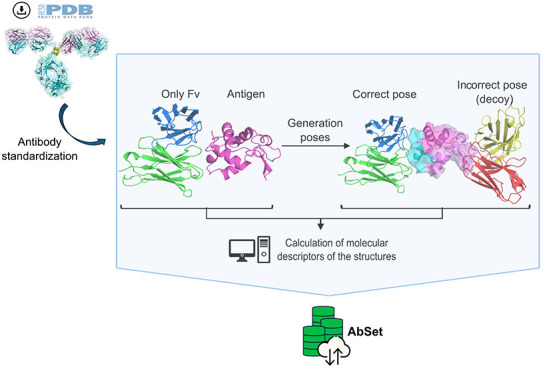

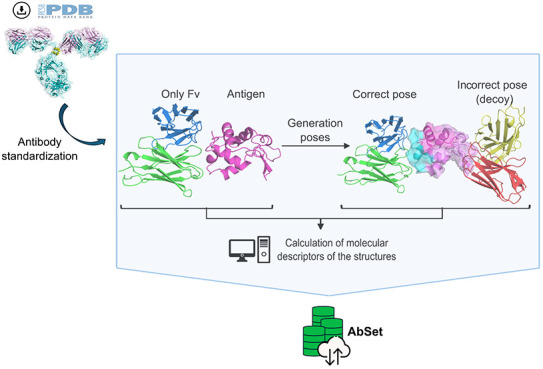

## 1 Introduction

Antibodies are potential therapeutic candidates for the development of safer and more effective therapies.^1^ These immune system proteins are produced by B lymphocytes in response to an antigen and have a Y-shaped structure composed of two light chains (VL) and two heavy chains (VH). They are divided into two main regions: the fragment antigen-binding region (Fab), which binds to and neutralizes the antigen, and the fragment crystallizable region (Fc), which interacts with the cell surface (). Antibody specificity occurs through variable regions (Fv), specifically through the three complementarity-determining region (CDRs) loops present in the variable domains of each light and heavy chain. Their components are designated CDR1, CDR2, and CDR3, whose conformation defines most of the paratope residues that directly interact with the antigen.^2^

The process of discovering and developing therapeutic antibodies has been optimized using computational methods, which provide a faster and more cost-effective alternative to traditional experimental techniques. Among these methods, machine learning algorithms have gained prominence and are being established in the pharmaceutical field. These algorithms consist of computational techniques to predict behaviors, values, and decisions, drawing from patterns learned in training data, with the quality of this data being a crucial factor in building robust AI models.^3,4^ Various databases of antibody sequences and structures are widely used to train these models, and structure-based methods are considered the most promising for antibody design.^5,6^ However, antibody structure data sets have limitations.

The first limitation is the availability of the data. Structural databases remain limited despite the millions of sequences in antibody databases, especially for antibodies complexed with protein antigens.^6^ Another challenge is the standardization of the structures. By nature, antibodies are diverse in their format, for example, scFv, VHH, and VH-VL. Furthermore, data extraction from the RCSB PDB database is not simple as there is no automatic way to recover all the antibody-containing entries that may contain multiple complexes or copies within the same file. Although AbDb provides standardized and numbered antibody structures for Fv, it is outdated. SabDab provides access to all structures retrieved from the PDB and offers numbering systems such as Chothia and IMGT. Additionally, its web interface features various search options, streamlining the process of screening for specific antibodies but still lacks consistent formatting for different antibody types.^7,8^ Finally, the antibody-specific structural databases integrated the molecular descriptors per residue, providing only PDB files with Cartesian atomic coordinates. However, enhancing these files with molecular descriptors and the physicochemical properties of each residue could improve the accuracy of protein–protein interaction models.^9^ Currently, none of the antibody-specific structural databases integrate molecular descriptors by residue.

Based on these limitations, we present AbSet. Our data set provides a comprehensive data set composed of standardized experimental structures of antibody–antigen complexes extracted from the RCSB PDB and represented through molecular descriptors at the residue level. The database was further enriched with an in silico generated subset for better data sampling, offering a robust and detailed resource for antibody–antigen interaction studies within a single curated data set.

## 2 Methods

The AbSet was developed through an automated routine in Python (Figure 1). Initially it retrieves and identifies experimental antibody structures from the RCSB PDB and further process it by numbering and standardizing antibody–antigen complexes. To improve the data diversity, an in silico subset was generated by performing molecular redocking and antibody modeling, producing variations in binding modes for each of the recovered complexes. Subsequently, molecular descriptors based on the surface and atomic characteristics were calculated. This process is detailed below.

**Figure 1:**
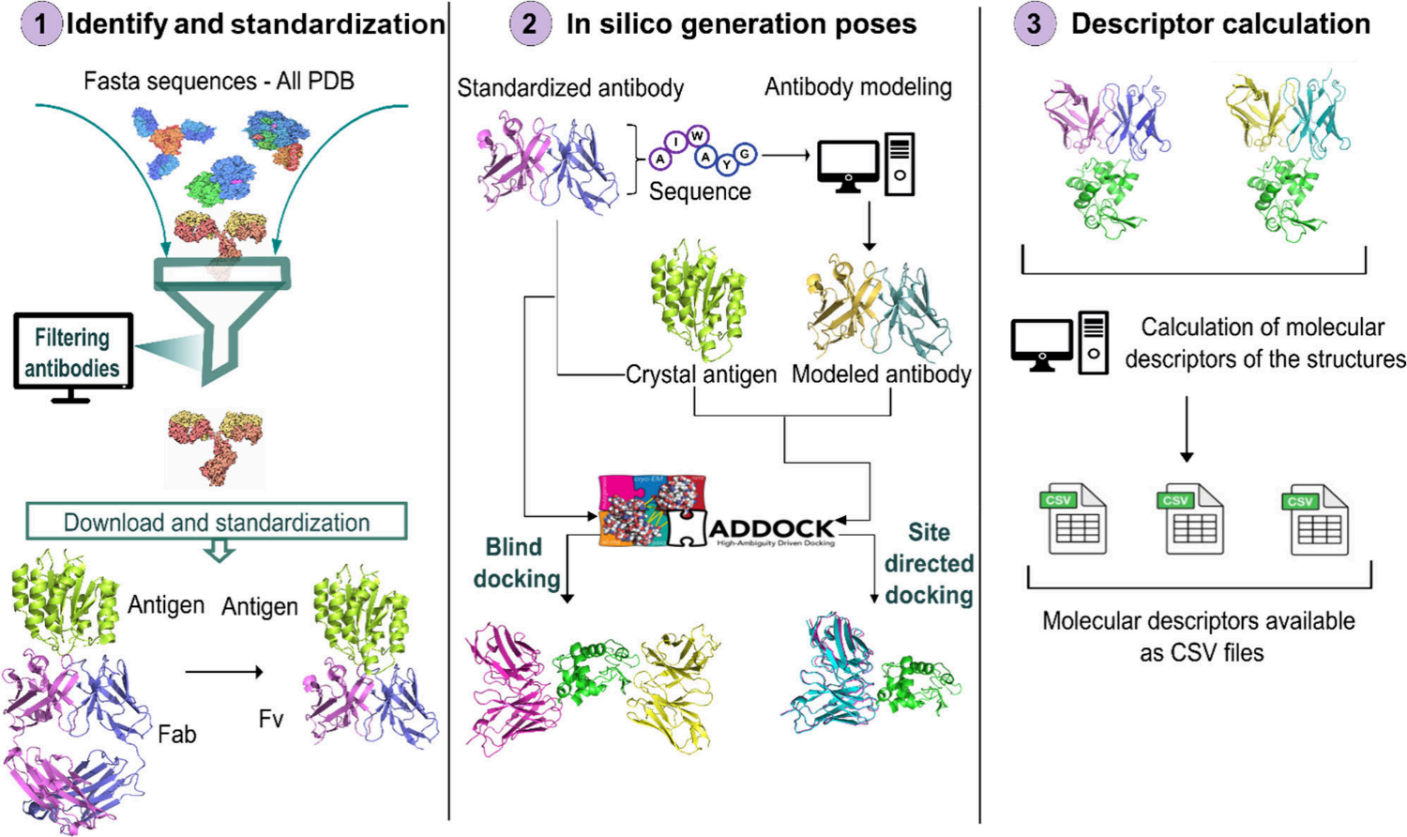
General automated flowchart of the AbSet. The process was automated using Python.

### 2.1 Processing of Experimentally Determined Structures

The first step in constructing AbSet was to retrieve the 3D structures of known antibodies complexed with protein antigens along with their experimental information. To achieve this, a file containing all available FASTA sequences from the PDB database was downloaded (accessed on November 20, 2024), which served as the foundation for this released data set. Using ANARCI, a specialized tool for numbering antibody amino acid sequences, the sequences were numbered according to the Martin scheme, restricting the analysis to antibodies only. The PDB IDs containing at least one sequence identified as a VH or VL antibody chain had their structure files, in .pdb format, downloaded for the next steps.^8^ In cases in which the PDB format was not available, the structures were downloaded in CIF format and subsequently converted to PDB.

A filter was applied to remove structures with missing atoms in amino acid residues and antibodies with unusual structures such as double variable domains. Each recovered structure was standardized by maintaining only the variable regions of the antibodies and ensuring one complex per file. The data set was divided into two subsets: (i) Free antibody, which includes all PDB IDs regardless of the presence of an antigen, and (ii) complex antibody, which contains only PDB IDs associated with protein antigens comprising more than 50 amino acid residues. To accomplish this, the following steps were implemented:

*Step 1: Renumbering the residues and renaming the antibody chains*. Antibody chains were identified, and their residues were renumbered according to the Martin scheme using ANARCI. The heavy and light chains of the antibodies were renamed H and L, respectively. In the case of scFv, the heavy and light chains were denoted as h and l, respectively. The antigen chains retain their original designations from the PDB file, except for those originally labeled as H or L, which are renamed chain “A”.

*Step 2: Identification of VH-VL pairs and antigens with biological interfaces.* To identify the true VH-VL chain pairs, the distances between the Cα atoms of Cys92 from VH and Cys88 from VL were measured. These residues are highly conserved and must be within a radius of 22 Å to be considered as part of the same antibody.^10^ For PDB entries containing multiple antigen chains, the interface between those chains was classified as biological or crystallization artifact using PRODIGY-CRIST, a set of scripts used to distinguish the interfaces between protein chains.^11^ If the interface is biological, the antigen chains are treated as oligomeric. Otherwise, the antigen chains with crystallographic interfaces were treated as a monomer.

*Step 3: Construction of antibody–antigen complexes.* Once the VH-VL pairings of the antibodies and the oligomeric state of the antigens are identified, it is necessary to determine which antibody and antigen entities form a biological interaction. To this end, the number of Cα contacts within 7.5 Å between the antigen residues and the CDRs was calculated. Complexes with at least one contact were identified as interfaces and were saved as PDB files. The resulting file was renamed as XXXX_n, where XXXX is the original 4-character PDB code and n is a counting flag to distinguish different biological complexes in the same PDB entry.

Using the obtained structures, a statistical profile of the AbSet was established, identifying the resolution and methods used for structure determination, diversity of antigens, and types of antibodies present. The resolution data and experimental methods were extracted directly from the PDB, whereas antigen diversity was determined using UniProt codes, which provided information about the proteins complexed with the antibodies. The parameters, including the experimental methods and resolutions, were retrieved from the corresponding PDB entries.

### 2.2 Construction of the In Silico Subset

The in silico subset was designed to increase the sampling of antibody–antigen binding modes and was developed using two main strategies. To build this subset, we selected a sample of antibody–antigen complexes with monomeric antigens. We tried to mimetize two common situations in computation development of antibodies; one starts with the crystallographic chains to generate complexes, and the second one includes the antibody modeling prior to the complex generation. Both samplings were generated by a docking process. The strategies aimed to generate a diverse set of binding modes, including correct, incorrect, and near-correct complexes, to better represent the variability in antibody–antigen interactions.

#### 2.2.1 Molecular Modeling

During the antibody modeling stage, the VH and VL chain sequences for each antibody were obtained from the FASTA files of the PDB database. Subsequently, these sequences were modeled using the ABodyBuilder2 model from ImmuneBuilder, excelling in predicting antibody structure and CDR conformation.^12^ The predicted structures were evaluated using the error estimate for each residue provided by the model itself for each residue of the CDRs.

#### 2.2.2 Molecular Docking

Redocking is a type of docking that involves analyzing the interaction mode between two molecules whose binding pose is already well-established. It was performed using the local installation of Haddock 2.4 software which is specifically designed for protein–protein docking calculations and has demonstrated good performance for antibody–antigen biological systems.^13^

The molecular docking process began with the preparation of biomolecules, starting with the numbering of the residues. A sequential numbering system was adopted to comply with the HADDOCK requirements to address the overlapping residue numbers in the two antibody chains. The heavy (H) and light (L) chains are treated with sequential numbering, but the light chain was renumbered to start at residue 500 to prevent overlap in residue numbering. Next, the active residues, defined by HADDOCK as those directly involved in the interaction, were identified. For antibodies, the active residues correspond to the amino acids located in the CDRs.

For crystallographic structures, a blind approach was used, considering all residues exposed on the molecular surface of the antigen to be passive. The solvent-accessible surface area was calculated using the FreeSAS C library; these residues, while not directly involved in the interaction, remain relevant for the docking process.^14^ This was part of a blind docking strategy in which no prior information about the binding site was used. In the case of modeled structures, the epitope residues on the antigen were defined as active based on changes in the solvent-accessible surface area between the complexed and uncomplexed antigen. In contrast to the previously described blind docking approach, a site-directed docking strategy was employed. The tautomeric/protonation states of the histidine residues were determined using the reduce module from the MolProbity software.^15^ The number of models generated during the randomization of orientations and rigid body minimization (it0), semiflexible refinement (it1) of the torsion angles ϕ, ψ, and ω, and explicit solvent refinement (itW) were defined as 1000, 250, and 250, respectively.

#### 2.2.3 Evaluation of Docking Results

The poses generated in silico were evaluated using the DockQ metric.^16^ DockQ combines a fraction of native interfacial contacts (Fnat), ligand RMSD (LRMS), and interface RMSD (iRMS) into a single score to assess docking quality. Based on this score, models are classified into four categories: high quality (DockQ ≥ 0.80), medium quality (0.49 ≤ DockQ < 0.80), acceptable (0.23 ≤ DockQ < 0.49), and incorrect (DockQ < 0.23).

### 2.3 Molecular Descriptors Calculation

Once the structures of the antibody–antigen complexes were standardized in both the experimentally derived and silico-generated subsets, molecular descriptors were computed to capture the characteristics of the amino acid residues and their surrounding environments. These descriptors were carefully selected to serve as suitable representations of the structures, enabling their use as input features for AI algorithms (Figure 2).

**Figure 2:**
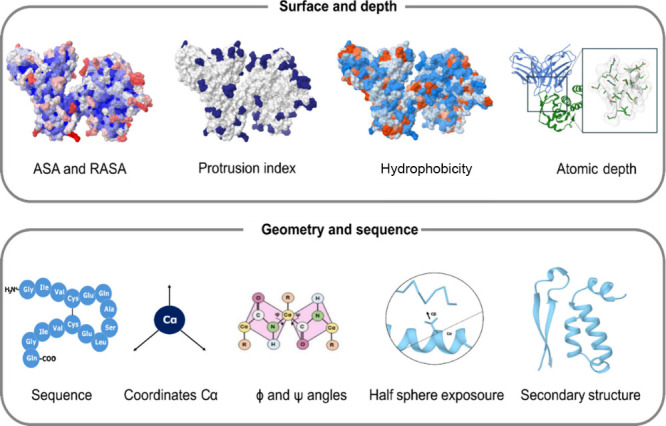
Molecular descriptors calculated for each residue of the structures in the AbSet data set.

The molecular surface characteristics were described by using both surface and volumetric information. The selected descriptors include the relative solvent-accessible area, atomic depth, protrusion index, and hydrophobicity, as they effectively capture the key properties of the amino acid residues and their environments. Additionally, other important descriptors were considered, such as the positions of the Cα atoms and the structural information on the proteins. These attributes were derived from half-sphere exposure calculations, Cα coordinates, ϕ and ψ dihedral angles, and the secondary structure of the protein.^17–24^

## 3 Results

### 3.1 Identification and Standardization of Antibodies

The analysis of the experimentally determined antibody structures involved the identification of relevant entries from the PDB database based on their FASTA sequences, resulting in a total of 9169 antibody entries. In contrast, SabDab, updated with the latest PDB version, reported 9026 antibody structures as of November 20, 2024. This revealed a discrepancy between the two data sets. To investigate this discrepancy, the entries identified by AbSet were compared with those retrieved by SabDab to determine structures that were uniquely present in either data set.

In total, 54 structures were identified in SabDab which were not identified in AbSet. All these cases are due to obsolete structures, such as 7CU4, and structures with their PDB codes replaced, such as 4nx3, which now has the code 4WEB. In contrast, 197 structures were identified using AbSet, which were not found in SabDab. Of these, only three structures were not classified as antibodies: two (7K0z and 7K0X) were identified as T-cell receptors (TCRs) and one (8thr) was classified as a human vesicular monoamine transporter. These were immediately removed. Specifically, PDB entry eighthr contains a sequence ranging between residues 745 and 858, which is highly similar to an antibody. These three structures were immediately removed from our data set. The remaining structures were antibodies, highlighting the need for careful curation of the process. A filter was also applied to remove structures with missing atoms, such as the 1IGA structure, which contains only Cα atoms, and antibodies with uncommon formats, such as PDB entry 4HJJ with a Dual Variable Domain, leaving 7449 structures.

These structures underwent standardization, resulting in 14184 PDB files with antibodies distributed among paired heavy and light chains (10495), single heavy chains (2852), single light chains (622), and scFv (215). Among these structures, 9146 are antibodies complexed with protein antigens, and 5038 are free antibodies. Antigens with multiple chains were constructed as described in the methodology section, to preserve their multimers. An example is the structure of PDB code 1MHP, which presents two antibody-integrin complexes, where the antigen chains establish only crystallographic contacts (Figure 3A). Consequently, two files were generated, each corresponding to a complex and one chain. The second example is structure 1BJ1, an endothelial growth factor homodimer that forms a biological interface, maintained in both files, each containing one antibody (Figure 3B).

**Figure 3:**
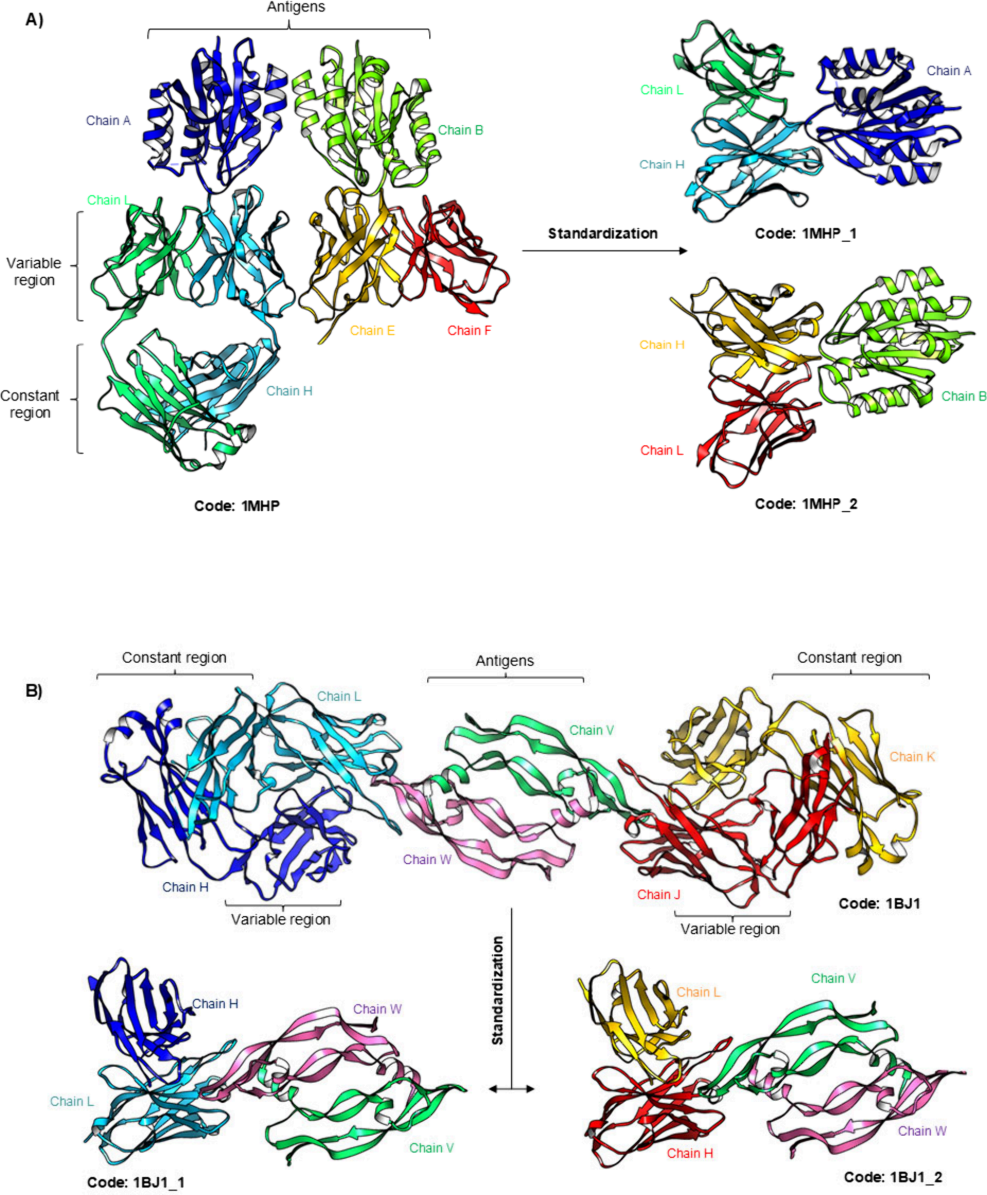
Standardization of the Antibody Structures. A) Example of standardization using the structure 1MHP, where two antigen chains form a crystallographic interface. The structure is depicted in cartoon form, with heavy chains shown in cyan and yellow, light chains in red and dark green, and antigens in lime green and blue. (B) Example using the structure 1BJ1, where two antigen chains form a biological interface. The structure is also represented in cartoon form, with heavy chains in blue and red, light chains in cyan, and antigens in lime green and pink.

#### 3.1.1 Crystallographic Resolution and Antigen Diversity of AbSet

Upon identifying the resolutions and methods used to determine the structures, it was found that 72.13% of the structures were determined by X-ray crystallography, 27.61% by electron microscopy, and 0.26% by other methods, such as NMR. The average resolution of the structures was approximately 2.4 and 3.7 Å for crystallography and electron microscopy, respectively. In addition, 86% of these structures had resolutions lower than 4.0 Å ().

Regarding the diversity of antigens, 21% of the antibodies were complexed with the spike glycoprotein of SARS-CoV-2 (UNIPROT entry P0DTC2). This protein plays a crucial role in viral interaction with host cells and serves as the primary target for immunotherapy during the COVID-19 pandemic.

### 3.2 In Silico Data set

We also constructed a second data set containing silico-generated complex structures using molecular docking. We selected a set of 1755 antibodies, extracted their FASTA sequences, and modeled their structures by using ABodyBuilder2. Subsequently, site-directed docking was performed with the crystallized antigen, which binds to the respective antibody. Additionally, we selected a set of 2135 experimentally determined antibodies complexed with antigens that were subjected to blind docking.

#### 3.2.1 Antibody Modeling

Upon evaluating the predicted root-mean-square error for the CDR residues, we identified that for antibodies with paired chains, the average model errors were 0.36 Å for CDRH1, 0.32 Å for CDRH2, 0.59 Å for CDRH3, 0.31 Å for CDRL1, 0.30 Å for CDRL2, and 0.34 Å for CDRL3. We observed some outliers, represented by structures with CDRH3 values exceeding 1 Å in both cases ().

#### 3.2.2 Molecular Docking

To increase the level of sampling of structures in the final set, we chose to adopt a strategy of generating new antibody–antigen poses, including biologically incorrect complexes. To achieve this, molecular docking of the modeled antibody–crystal antigen complexes generated 438750 new binding poses, classified according to DockQ metrics as 308888 incorrect, 45800 acceptable, 66796 medium, and 17266 high-quality. Regarding the complexes formed with both the experimentally determined antigen and antibody, 438750 binding poses were generated, classified as 520125 incorrect, 3938 acceptable, 5639 medium, and 4048 high quality.

In high-quality, medium-quality, and acceptable models, there was a significant overlap with the crystallized antibody, accompanied by only minor changes in the paratope orientations. This characteristic was not observed in incorrect models, in which the antibody paratope interacted with an epitope distinct from the original antigen (). These incorrect poses are particularly valuable in enriching the AbSet data set, as they can serve as a set of decoys for incorrectly interacting antibody–antigen complexes.

### 3.3 Implementation of Molecular Descriptors

For AbSet, molecular descriptors were calculated for all standardized antibody structures retrieved from the PDB. For the structures generated in silico through docking, molecular descriptors were computed for four structures from a set of 250 poses generated for each system. These 4 structures represent one from each category, as assessed using DockQ. However, for systems that failed to generate poses in all categories, incorrect poses were included to complete the subset of 4. Molecular descriptors were stored in a CSV file, with columns representing the attributes and rows corresponding to the sequence of amino acid residues.

To evaluate the descriptor calculation performance, we selected 16 structures as examples. The average execution time of the program using the 6 processors remained stable as the number of processed PDB files increased. Execution times ranged from 10 s for a single file to 40 s for all PDB files, demonstrating proportional growth with the data volume (). Tests were conducted on an Intel model W-2135 processor. Memory usage was not a limiting factor, given the relatively short execution time.

## 4 Discussion

The retrieval of antibodies from the PDB and the use of ANARCI for numbering are well-established practices that have been widely utilized in various applications.^25–28^ However, one challenge is the standardization of structures, specifically retaining only the variable region of the antibody, a resource that remains somewhat limited in the literature. AbNum is a primary software tool designed to perform this standardization, which returns structure files directly. It is available both as a server and as a Python API;^29^ it offers useful functionality but also presents certain limitations, particularly when processing unusual structures. For instance, while AbNum generally performs well, it may struggle with specific cases, such as identifying chains correctly in certain antibody structures or handling more uncommon variations, as highlighted by Martin et al.^7^

Our approach addressed these gaps by offering a more robust solution. The standardization script developed for the AbSet database uses an ANARCI-numbered file as its foundation to generate an accurate new PDB file. Unlike other tools, our script successfully handles unusual antibody structures, including those that may not be recognized by AbNum, ensuring comprehensive coverage of the antibody variants. This approach complements the existing algorithms by overcoming their limitations and providing a more flexible and inclusive solution for antibody structure standardization.

Furthermore, algorithms that use structural data for training AI models often apply a resolution filter, typically setting an average threshold around 3.5 Å.^25,30,31^ Notably, 86% of the structures in AbSet fell within this range with a resolution better than 4 Å. This is a critical factor for ensuring the quality and accuracy of the structural data used in AI-based model training. The average resolution of the AbSet structures was 3.3 Å, which aligns with the refinement values reported by Casadio et al., who indicated that structures with resolutions of up to 4 Å still provide reliable atomic representations.^32^

Turning to the diversity of antigens in the AbSet database, we observed a notable prevalence of antibodies complexed with SARS-CoV-2. This finding aligns with extensive research efforts during the COVID-19 pandemic, which focused on developing antibodies targeting the virus.

The modeled antibodies demonstrated good reliability, with ABodyBuilder2 showing high efficacy, with only minor variations in the CDRH3 loop, a common feature owing to its inherent variability.^30^ ABodyBuilder2, recognized as state-of-the-art for antibody modeling, has been used to model approximately 1.5 million sequences from the Observed Antibody Space (OAS) database.^12^ These modeled antibodies, along with experimentally determined structures, were subjected to molecular docking calculations, revealing different binding poses and generating a refined set of decoys and incorrect antibody–antigen interactions, thus enriching the data volume of AbSet. Each structure was associated with a DockQ score that classified the complexes into four quality levels: high, medium, acceptable, and incorrect (decoy). The DockQ score ensures that each pose is viable while also reflecting perturbations in the experimental structure. This introduces diversity into the data set, enabling the training of machine learning models with data that closely resemble real scenarios.

We took an additional step by developing a script that calculated the molecular descriptors for all of the structures in AbSet. This script, designed for efficient execution, is publicly available to enable other researchers to apply it to diverse protein systems.

## 5 Conclusion

In this study, we present AbSet, a highly standardized data set of antibody structures, including both variable regions and interacting antigens, enriched with a vast array of data, such as molecular descriptors and decoys representing diverse binding modes. These structures are meticulously curated to reflect key biochemical properties at the residue level. AbSet provides a valuable resource for the training and optimization of machine learning models in antibody discovery. Furthermore, the data set and accompanying software tools for molecular descriptor calculations will be made publicly available to support further research in this field.

## Supplementary Material





## Data Availability

The AbSet is available in a repository on Zenodo (https://doi.org/10.5281/zenodo.14888002), which includes standardized PDB structures, in silico generated structures, and CSV files containing molecular descriptors. Additionally, we have created a GitHub repository for calculating molecular descriptors (https://github.com/SFBBGroup/AbSet.git), which also includes a script for filtering PDB files based on a specific resolution threshold from a PDB file.

## Notes

Supplementary_additional_results.xlsx - Tables listing all retrieved antibody structures from the PDB, unique antibodies in AbSet, and unique antibodies in SAbDab (XLSX)
Supplementary_additional_figures.docx - Figures on antibody structure, resolution distribution, CDR modeling error, docking pose overlap, descriptor calculation processing time, and one table comparing AbSet with other data sets (PDF)

## Notes

All contributions by Geraldo Rodrigues Sartori were made entirely during his institutional affiliation with the Fundação Oswaldo Cruz, FIOCRUZ. The author is currently affiliated with the Instituto de Física de São Carlos, Universidade de São Paulo, IFSC/USP

## Abbreviations

AbSet: Antibody data set
VL: light chains
VH: heavy chains
Fc: crystallizable region
Fv: variable regions
CDR: complementarity-determining region
scFv: single-chain variable fragmente
PDB: protein data bank
Fnat: native interfacial contacts
LRMS: ligand RMSD
iRMS: interface RMSD
TCR: T-cell receptors
