# Supplementary material for: AbSet: A Standardized Data Set of Antibody Structures for Machine Learning Applications
Source: J Chem Inf Model. 2025 May 11;65(10):4767–74. doi: 10.1021/acs.jcim.5c00410 (PMC12117563; doi:10.1021/acs.jcim.5c00410)
Supplement: Supplementary file 2 [file ci5c00410_si_002.pdf]

ABSET: A STANDARDIZED DATASET OF ANTIBODY STRUCTURES FOR MACHINE LEARNING APPLICATIONS

Diego S. Almeida[1,2]; Matheus V. Almeida[1]; Jean V. Sampaio[1,2]; Eduardo M. Gaieta[1,2]; Andrielly H. S. Costa[1,2]; Francisco F. A. Rabelo[3]; João E. Lemos[1,3]; César L. Cavalcante[3]; Geraldo R. Sartori[1]*; João H. M. Silva[1,2,3]*.

[1]Laboratory of Structural and Functional Biology Applied to Biopharmaceuticals, Fundação Oswaldo Cruz, Fiocruz Ceará, Eusébio, Brazil.

[2]Instituto Oswaldo Cruz, Fiocruz, Rio de Janeiro, Rio de Janeiro, Brazil.

[3]Universidade Federal do Ceará, Fortaleza, Brazil.

[4]Pasteur-Fiocruz Center on Immunology and Immunotherapy, Fundação Oswaldo Cruz, Fiocruz Ceará, Eusébio, Brazil.

SUPPORTING INFORMATION

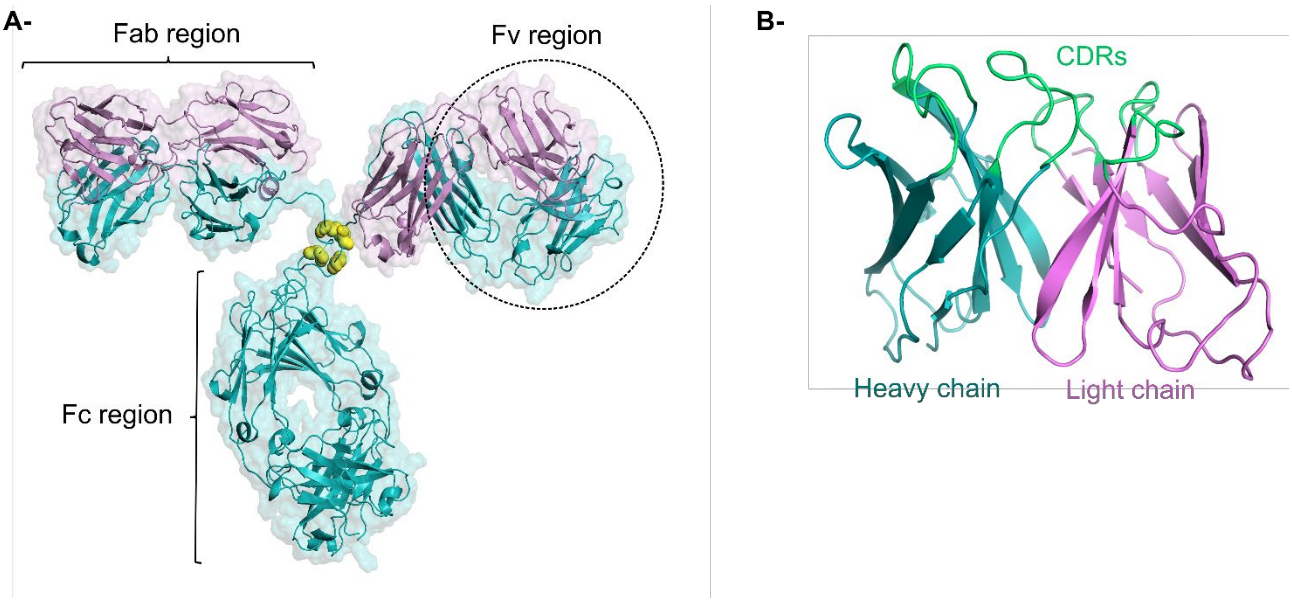

Figure S1 – Antibody structure. A – Overall structure of the antibody illustrating the heavy (teal) and light (violet) chains. B – Variable domain of the antibody, with emphasis on the CDRs, highlighted in shades of grenn. PDB ID: 1igt.

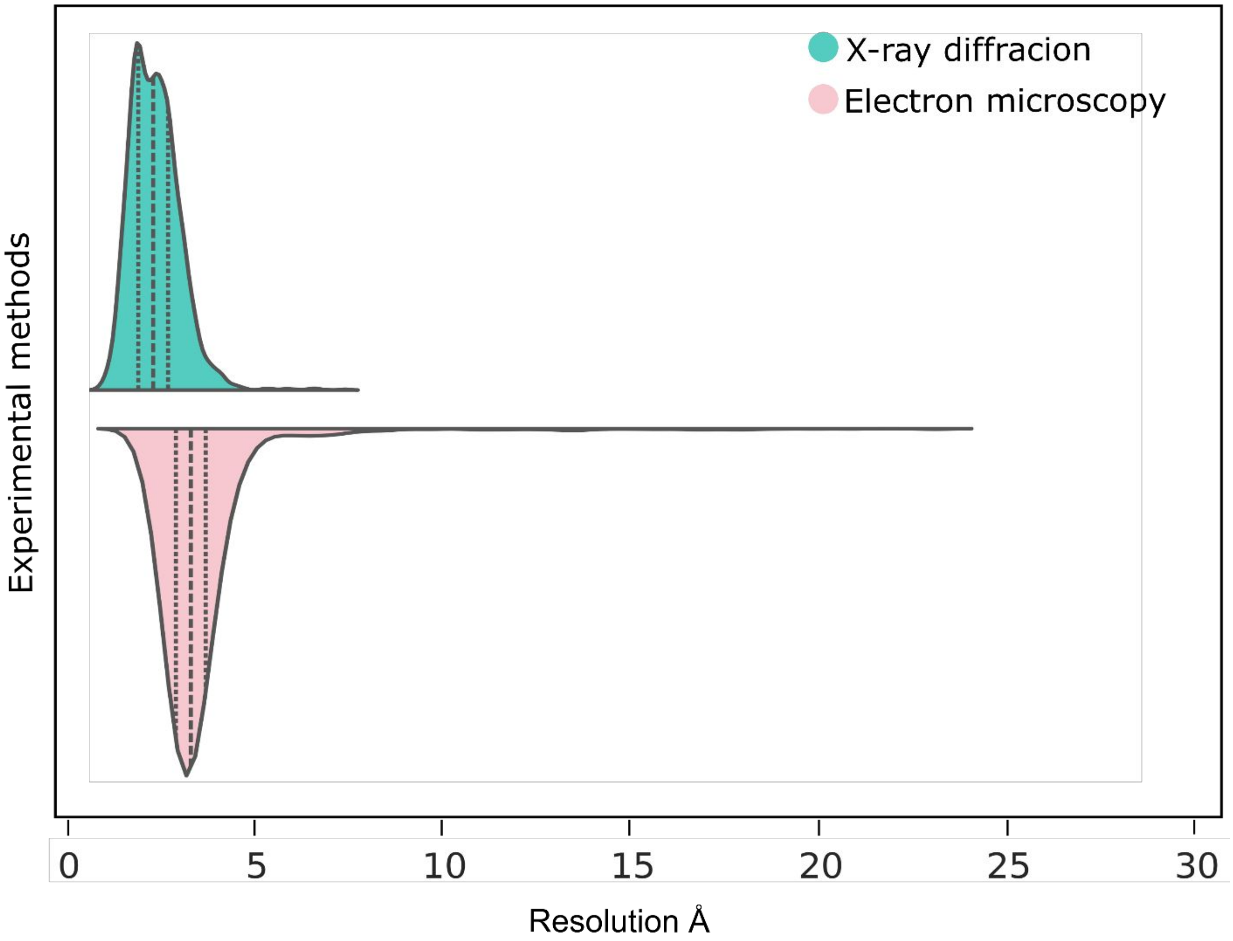

Figure S2 - Distribution of resolutions for experimentally determined structures, with X-ray diffraction structures shown in purple and electron microscopy structures in yellow.

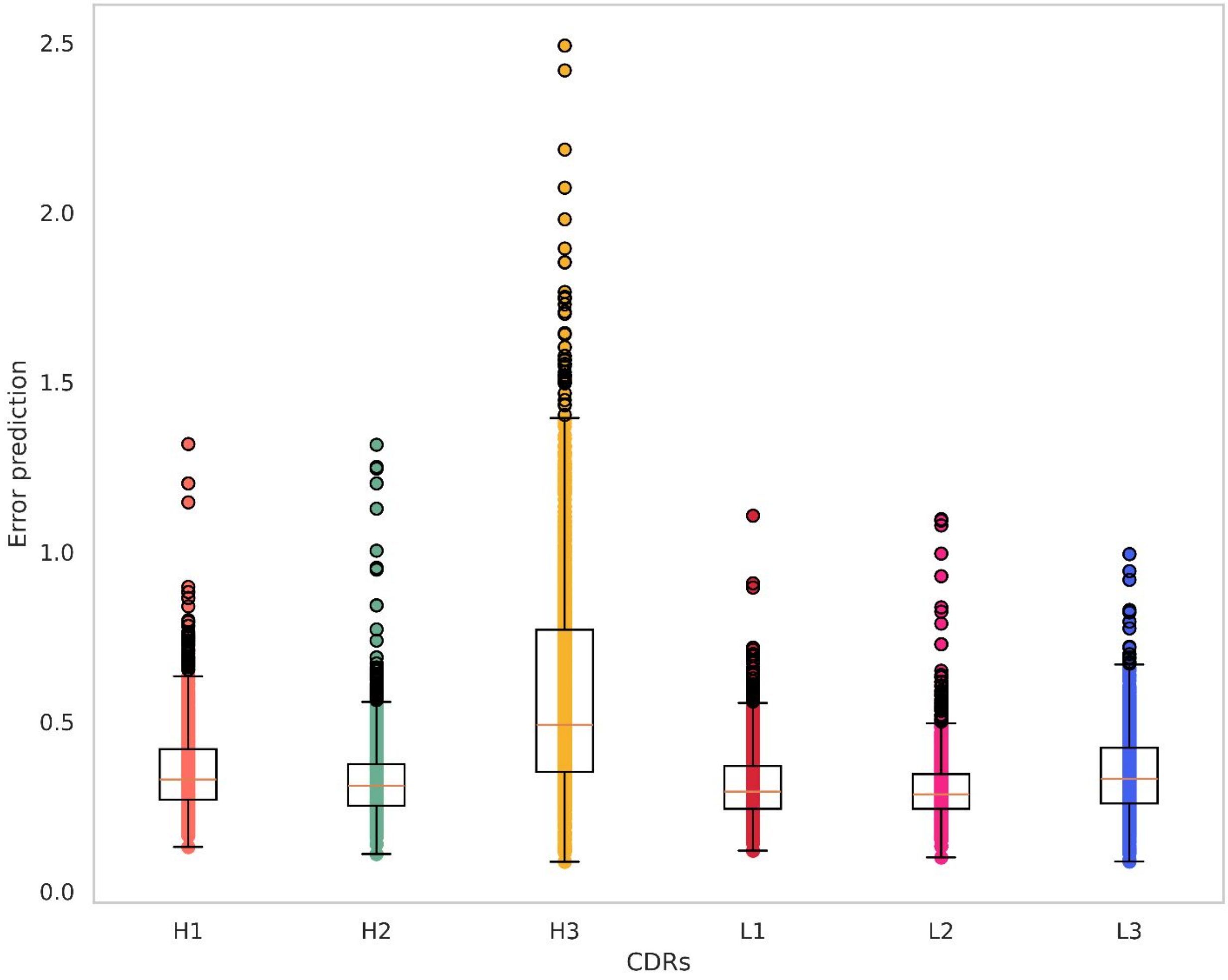

Figure S3 – Evaluation of the predicted average error for each CDR in the modeled structure set using ABodyBuilder2.

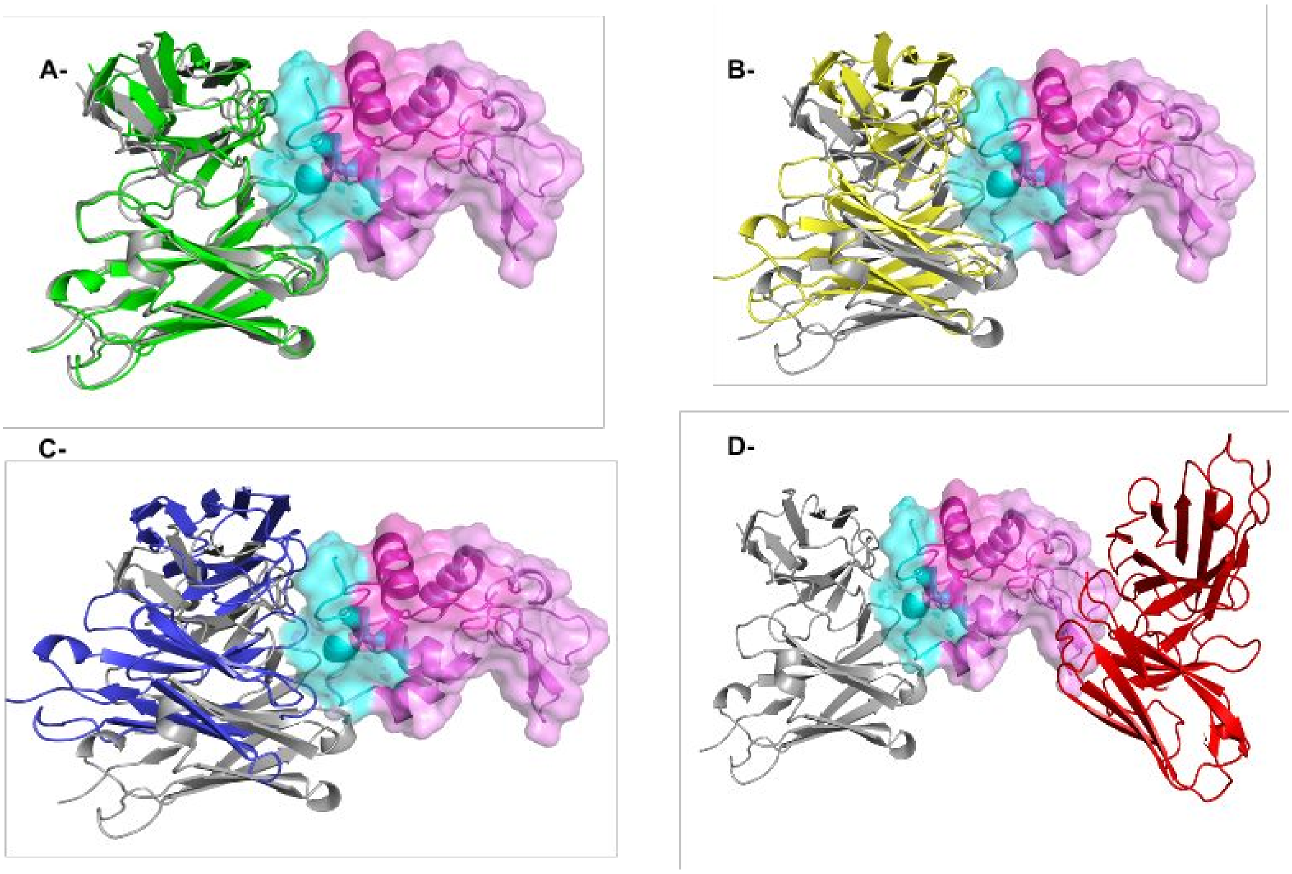

Figure S4 – Overlap of docking poses with the experimentally determined antibody (gray) in the DockQ classifications. The antigen is represented as a magenta-colored surface, with its epitope highlighted in cyan. A – High quality pose (DockQ = 0.819). B- Medium quality pose (DockQ = 0.751). C - Acceptable pose (DockQ = 0.367). D – Incorrect pose (DockQ = 0.03).

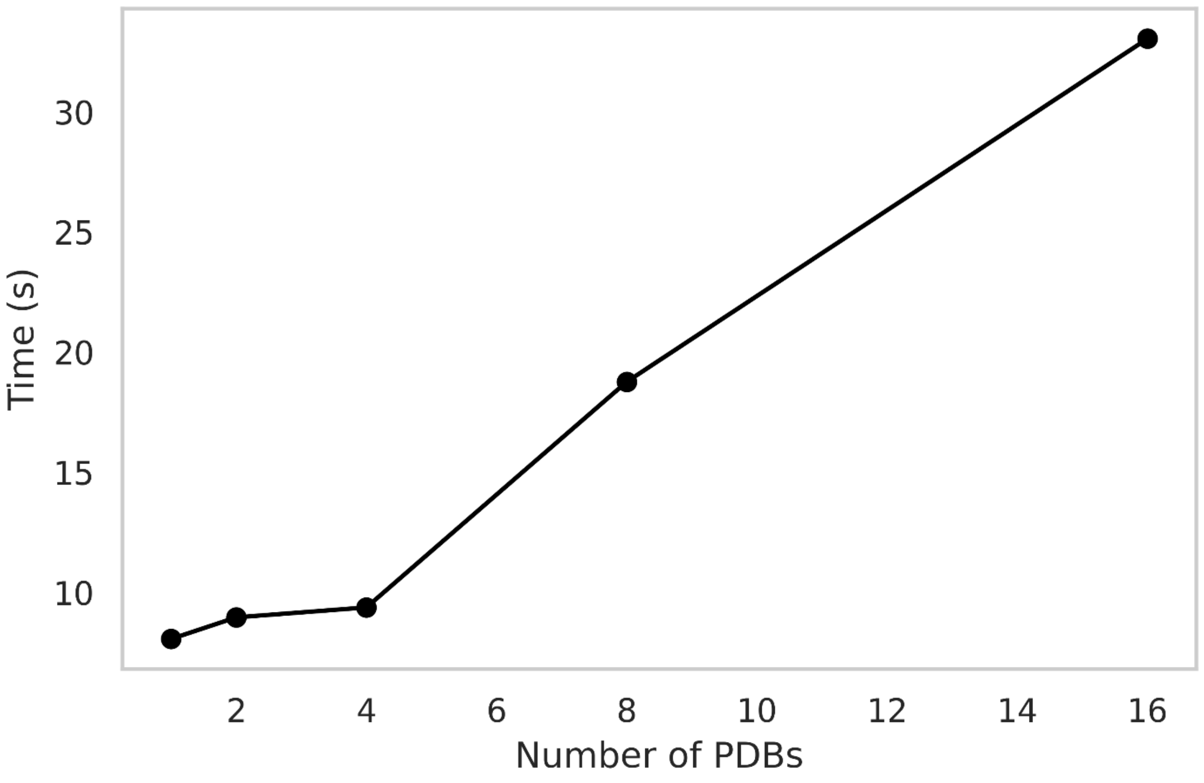

Figure S6 – Average processing time (in seconds) for different numbers of PDBs. The plot shows the mean time for each process, with a logarithmic scale on the x-axis to better represent the exponential growth in PDB count.

Dataset / Database	Web interface	Standardized antibodies	Molecular descriptors	self-updating	Decoy set	Reference
**AbSet**	No	Yes	Yes	No	Yes	-
**SabDab**	Yes	No	No	Yes	No	[1]
**AbDb**	Yes	Yes	No	No	No	[2]
**Thera-SAbDab**	Yes	No	No	Yes	No	[3]
**CoV-AbDab**	Yes	No	No	Yes	No	[4]
**NanoLAS**	Yes	No	No	Yes	No	[5]
**PLAbDab**	Yes	No	No	Yes	No	[6]

Table 1 – Comparison of the AbSet with current antibody structure databases.

REFERENCES

(1) Dunbar, J.; Krawczyk, K.; Leem, J.; Baker, T.; Fuchs, A.; Georges, G.; Shi, J.; Deane, C. M. SAbDab: The Structural Antibody Database. *Nucleic Acids Res* 2014, *42* (D1). https://doi.org/10.1093/nar/gkt1043.

(2) Ferdous, S.; Martin, A. C. R. AbDb: Antibody Structure Database - A Database of PDB-Derived Antibody Structures. *Database* 2018, *2018* (2018). https://doi.org/10.1093/database/bay040.

(3) Raybould, M. I. J.; Marks, C.; Lewis, A. P.; Shi, J.; Bujotzek, A.; Taddese, B.; Deane, C. M. Thera-SAbDab: The Therapeutic Structural Antibody Database. *Nucleic Acids Res* 2020, *48* (D1), D383–D388. https://doi.org/10.1093/nar/gkz827.

(4) Raybould, M. I. J.; Kovaltsuk, A.; Marks, C.; Deane, C. M. CoV-AbDab: The Coronavirus Antibody Database. *Bioinformatics* 2021, *37* (5), 734–735. https://doi.org/10.1093/bioinformatics/btaa739.

(5) Xiong, S.; Liu, Z.; Yi, X.; Liu, K.; Huang, B.; Wang, X. NanoLAS: A Comprehensive Nanobody Database with Data Integration, Consolidation and Application. *Database* 2024, *2024*. https://doi.org/10.1093/database/baae003.

(6) Gordon, G. L.; Greenshields-Watson, A.; Agarwal, P.; Wong, A.; Boyles, F.; Hummer, A.; Lujan Hernandez, A. G.; Deane, C. M. PLAbDab-Nano: A Database of Camelid and Shark Nanobodies from Patents and Literature. https://doi.org/10.1101/2024.07.19.604232.
